# Does variation in egg structure among five populations of Atlantic salmon (*Salmo salar*) influence their survival in low oxygen conditions?

**DOI:** 10.1098/rsos.181020

**Published:** 2019-01-30

**Authors:** Jack Bloomer, David Sear, Paul Kemp

**Affiliations:** 1 Department of Geography and the Environment, University of Southampton, Building 44, University Road, Southampton SO17 1BJ, UK; 2 International Centre for Ecohydraulics Research, Faculty of Engineering and the Environment, University of Southampton, Highfield, Southampton SO17 1BJ, UK

**Keywords:** chorion, permeability, hypoxia, adaptation, tolerance

## Abstract

Oxygen supply to the salmonid egg surface can be limited by external factors such as sedimentation and groundwater upwelling, while the egg membrane itself can impede diffusion from the egg surface to the embryo. Therefore, the structure of egg membranes could affect the rate at which embryos obtain oxygen from their surroundings. Published field data indicate that oxygen stress experienced by salmonid eggs can vary widely among populations. Therefore, if membrane architecture influences diffusion rate to the embryo, selection for more permeable membranes could occur in oxygen-stressed environments. Using electron microscopy, the membrane structure of eggs obtained from five UK Atlantic salmon (*Salmo salar*) populations is described. Membrane thickness, porosity and permeability to dissolved oxygen varied among populations. Furthermore, comparison of membranes of eggs that survived laboratory controlled low-oxygen conditions compared to those that died suggested that ova with less permeable membranes were more susceptible to hypoxia-induced mortality. In addition, membrane porosity was lower than previously reported indicating that oxygen requirements during incubation have been underestimated, so models such as the mass transfer theory that predict incubation success could currently overestimate ova survival. Variation in egg membrane structure influences low oxygen tolerance of Atlantic salmon embryos and could represent adaptation to low oxygen stress. Consequently, stock enhancement techniques such as supportive breeding that relieve incubation stress could erode structural adaptations.

## 1. Introduction

During the incubation stage of their life cycle, oviparous organisms are exposed to a range of stressors that can play a crucial role in shaping individual fitness and population dynamics. Their immobile nature means eggs cannot escape these stressors, so the incubation environment determines the nature of the hazards they face. While the nature of hazards faced by oviparous organisms varies throughout the animal kingdom, benthic spawning teleosts such as salmonids frequently suffer mortality through predation [1], fungal and bacterial colonization [2], siltation [3] and hypoxia [4]. By burying their eggs in nests in riverbed gravels known as redds [5], the vulnerability of salmonid ova to predation is reduced. However, this action can increase the susceptibility of eggs to microbial and fungal infection, siltation and hypoxia [6].

Although the nature of oxygen delivery to eggs of oviparous organisms varies enormously depending on the life cycle and incubation conditions of the species in question, it can become limiting to survival and post-hatch fitness when embryonic demand outstrips supply [7]. In the case of Atlantic salmon (*Salmo salar*), oxygen is supplied to the embryo from a thin film of water surrounding the egg known as the boundary layer [8]. The rate of supply to the boundary layer is greatest when the interstitial velocity and oxygen concentration of water flowing past the eggs is high, so is influenced by factors such as fine sediment concentration in redds [8] and groundwater upwelling [9] which reduce stream bed permeability and oxygen concentration respectively. In addition, oxygen demand is greater at higher temperatures [10], so vulnerability to oxygen stress is likely to be greatest in warmer conditions. While factors such as fine sediment load, groundwater input and temperature vary significantly within river systems [4,8], field data show that this variation is much greater among river systems that host different Atlantic salmon populations in the UK [8,9,11]. Therefore, different populations of Atlantic salmon in the UK are likely to experience different levels of oxygen stress during the incubation stage of their life cycle.

While external factors influence oxygen supply and demand, the protective membrane that surrounds the embryo can impede diffusion and exacerbate hypoxic stress [12]. Salmonid egg membranes are composed of a dense layer of proteinaceous fibres, interspersed with micropores [13]. The fraction of the egg surface composed of these micropores determines membrane porosity and, along with membrane thickness, controls total permeability to a range of substances including dissolved oxygen [14,15]. As a result, eggs with greater membrane thickness or lower porosity could be more likely to experience oxygen stress.

The importance of porosity and thickness in terms of oxygen supply to embryonic salmonids is analogous to the egg membranes of oviparous organisms. Indeed, studies have shown that other species demonstrate inter-population variability of egg architecture in response to oxygen availability [16]. Thus, we hypothesize egg architecture will vary among Atlantic salmon populations according to the frequency and degree of oxygen stress that they face during incubation as part of an adaptive compensatory response. Population variation in the response of Atlantic salmon eggs to hypoxia has been observed and attributed to differences in the physiological response of the embryo [17]. However, the authors did not consider inter-population differences in oxygen uptake rates, which could be driven by structural variance of the egg membrane. If structural differences among populations are present and affect tolerance to oxygen stress, then there would be important implications for stock enhancement programmes that rear eggs of wild fish in a hatchery environment. In addition, deterministic models such as the mass transfer theory [7] and the sediment intrusion and dissolved oxygen transport model (SIDO) [18] that predict ova survival based on incubation conditions assume uniform membrane permeability across populations. If structural differences exist, the application of such models to a range of populations may need to be re-visited, as they can play an important role in guiding catchment and hydrological management practices [19,20].

This study aimed to determine whether Atlantic salmon populations exhibit distinct egg architecture and whether egg structure varies alongside embryonic sensitivity to hypoxia. These aims were achieved through three specific objectives: (1) determine inter-population variability of egg architecture of five Atlantic salmon populations, with a particular focus on permeability to oxygen by using scanning electron microscopy to measure key structural features; (2) determine the effect of differences in egg architecture on estimated in-redd conditions required to support ova respiration by applying the measurements obtained from objective 1 to the mass transfer model; and (3) determine whether there was a difference in egg architecture between eggs of a single population that survived extended periods of hypoxia, and those that died.

## 2. Material and methods

### 2.1. Objective 1: Inter-population variability of egg architecture

**Figure 1.**
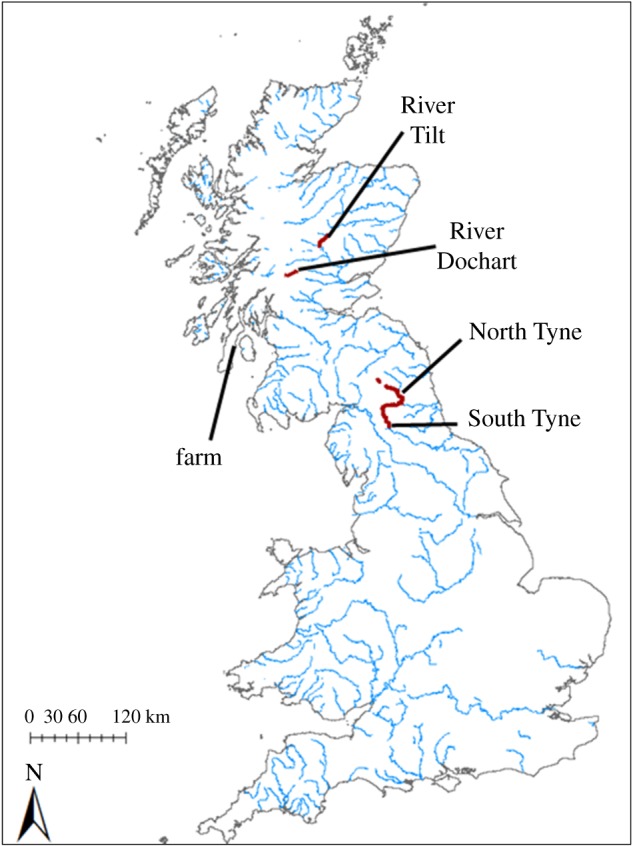
Map of United Kingdom with rivers hosting Atlantic salmon shown in blue. The locations of the egg sources used in this study are labelled and displayed in heavy red.

Gametes were sourced from five different Atlantic salmon populations throughout the United Kingdom (figure 1). Four of these (River Dochart, River Tilt, River South Tyne, River North Tyne) were taken from conservation hatcheries where reproductively mature wild caught Atlantic salmon were captured from their natal streams during peak spawning season. The fifth population was sourced from a commercial hatchery that supports the aquaculture industry. Eggs of the farmed population were originally sourced from adults of Norwegian origin, but have been bred entirely in captivity for more than 10 generations.

For each population eggs were extracted from 10 female fish and milt from 4 male fish (table 1), and the gametes of each parent fish were stored separately. Following extraction, eggs and milt were placed in well-oxygenated plastic bags (eggs submerged in coelomic fluid) and transported to the University of Southampton in chilled polystyrene containers where they were fertilized within 24 h of the gamete extraction procedure.

**Table 1.** Details of the rivers and hatcheries from which eggs were sourced for the current investigation in order to meet all research objectives. The date of fertilization indicates the time at which incubation of each population began in the University of Southampton research facility. Note: Unless otherwise stated, information is based on the hatchery from which eggs were sourced.

source river	hatchery location	hatchery location (lat, long)	hatchery altitude (m.a.s.l.)	mean incubation temperature (°C)	mean maternal mass (kg)	stripping date	fertilization date
River Dochart	Perthshire, Scotland	56.41, −3.47	12.42	3.00	5.15 ± 0.40	10 Nov 2015	11 Nov 2015
River Tilt	Perthshire, Scotland	56.42, −3.47	12.42	3.00	5.01 ± 0.45	10 Nov 2015	11 Nov 2015
farm	Argyll and Bute, Scotland	55.89, −5.62	6.47	4.00	5.48 ± 0.50	16 Nov 2015	17 Nov 2015
River South Tyne	Northumberland, England	55.23, −2.58	210.10	5.12^a^	4.54 ± 0.32	2 Dec 2015	3 Dec 2015
River North Tyne	Northumberland, England	55.23, −2.58	210.10	4.37^a^	4.29 ± 0.44	2 Dec 2015	3 Dec 2015

^a^Temperature data sourced from Environment Agency (2007) for mean river temperature.

Gametes were crossed using a full-factorial breeding design [21] for each population wherein milt from each male was paired with eggs from each female, resulting in 40 unique families per population. To create each family, the eggs of each female were divided into four groups of 30. Each group of 30 eggs was combined with 0.1 ml milt from a different male of the same population and mixed by hand for 60 s in a dry container. Fertilized eggs were subsequently washed and allowed to harden for 1 h using water from the experimental facility [22]. This procedure was repeated simultaneously for each population, resulting in 200 families in total (40 families × 5 populations). Due to space constraints, five eggs from each family were subsequently selected at random and pooled into a single group with eggs of the same population, resulting in a group of 200 eggs for each population (40 families × 5 eggs). Eggs of each population were placed into a single Perspex chamber (25 × 15 × 12 cm, water depth 7 cm) lined with artificial grass [23] immediately after the fertilization procedure was complete. While the process of combining eggs from each maternal fish into a single group per population was necessary due to space constraints, the process had the potential to give rise to pseudo-replication whereby individual parent fish could be over-represented in the data. This risk was limited by ensuring eggs were well-mixed within the egg chamber and a high number of replicates per population (36) were sampled to maximize the spread of data sources.

Oxygen-saturated water flowed through the chambers at a bulk rate of 150 cm h^−1^ ± 3.4%. Before entering the egg chambers, the water was exposed to chemical, biological and physical treatment to ensure it was suitable for rearing of Atlantic salmon ova [24].

Ambient temperature plays a key role in determining developmental rate of incubating salmonids [10]. Therefore, to determine the developmental state of embryos throughout this investigation, water temperatures were continuously recorded throughout the experiment using Aandera^®^ optodes and a Delta-T data logger. The optodes recorded temperature at 1 min intervals and the 10 min mean was stored on the data logger. The recorded 10 min mean temperature values enabled calculation of the daily embryonic developmental state in degree days (DD) using the following formula:

$$\mathrm{DD}=\;\frac{T}{144},$$

where DD = developmental rate of the embryo over each 10 min reading, referred to as degree days; 144 = number of 10 min readings taken in a 24 h period; *T* = temperature (°C).

The above calculation was conducted every 24 h and a cumulative value calculated to provide the developmental state of embryos on a daily basis. Subsequently, eggs from each population were sampled at 100 DDs. Sampling at the same developmental state for each population ensured that possible changes in structure during development [21] did not unequally affect samples.

### 2.2. Objective 2: Mass transfer model

The mass transfer theory [7] enables theoretical estimation of the oxygen supply requirements of incubating ova according to key variables of oxygen concentration, water velocity past the egg surface and egg architecture. Here, the model was used to calculate the lower limits of oxygen concentrations at fixed water velocities (1, 5, 10, 50, 100, 250, 500, 750, 1000, 5000 and 10 000 cm h^−1^) necessary to support incubating salmonids of each population according to their measured variation in egg architecture. As such, the effect of egg structure on the critical oxygen threshold could be calculated as

**Figure 2.**
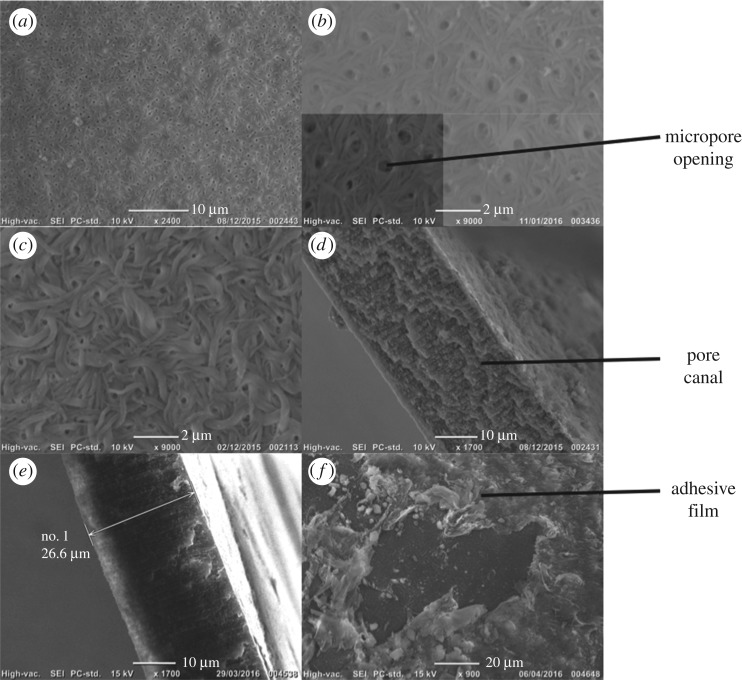
Images of membrane structures used in analysis: (*a*) regular micropore arrangement on membrane internus at 2400× magnification; (*b*) membrane internus with micropore dimensions at 9000× magnification and lower quadrant where measurements of micropore diameter were taken is highlighted; (*c*) membrane internus at 9000× magnification; (*d*) membrane cross-section traversed by pore canals at 1700× magnification; (*e*) membrane cross-section with thickness dimensions; (*f*) image of the membrane externus where the adhesive film is present at 900× magnification. An area where the film is missing, revealing the membrane itself can be observed in the centre of the image.

2.1 $$C_{1}=\;\;\frac{N_{\mathrm{req}}[\left(1/k)+(x/D_{c}\right)]}{4\pi r^{2}+C_{e}},$$

where *C*_1_ = required oxygen concentration of water within redd to prevent metabolic inhibition; *N*_req_ = oxygen consumption of embryo (mg s^−1^); *k* = mass transfer coefficient (cm^2^ s^−1^), calculated from a formula taken from the literature [25]; *x* = membrane thickness (cm), based on mean value for each population; *r* = egg radius (cm), based on mean value for each population; *C*_e_ = oxygen concentration within perivitelline fluid of the egg (1.1 × 10^−3^ mg cm^−3^) taken from rainbow trout (*Oncorhynchus mykiss*) embryos [26] as values for Atlantic salmon were unavailable. *D*_c_ (membrane diffusivity, cm^2^ s^−1^), based on the mean value for each population was calculated using

$$D_{c}=\;\frac{D\varepsilon_{\tau }\delta }{\tau },$$

where *D* = Diffusion coefficient of oxygen in water (cm^2^ s^−1^); ɛ*_τ_* = membrane porosity (dimensionless); *δ* = constrictivity; *τ* = tortuosity. Based on observation of pore canals in figure 2*d*, constrictivity and tortuosity were assigned a value of 1 due to their relatively straight nature.

Previous application of the mass transfer model relied on porosity and thickness estimates of Chinook salmon (*Oncorhynchus tshawytscha*) and brown trout (*Salmo trutta*) eggs presented in [7] and [25] respectively. Here, the output of the model using these data (radius = 0.3 cm; thickness = 3.5 × 10^−5^ cm; porosity = 0.1) was compared with the mean values of porosity and thickness for each population of Atlantic salmon studied. All data were compared using values calculated at 5°C to ensure consistency.

### 2.3. Objective 3: Egg structure and hypoxic tolerance of farmed eggs

To determine if structural variation of egg membranes corresponded with differences in embryonic tolerance to hypoxia, a total of 2400 additional eggs were taken from the 10 maternal fish (240 eggs per fish) of the farmed population and fertilized following the protocol described above (§2.1). These eggs were subsequently split into two treatment groups (control and hypoxic) of 1200. The control treatment received oxygen-saturated (mean: 10.65 ± 1.43 mg l^−1^) water throughout development, while the mean concentration in the hypoxic treatment was 1.56 ± 0.49 mg l^−1^. Oxygen levels were controlled through a process of nitrogen sparging following methods described elsewhere [24]. Only farmed eggs were studied to ensure possible inter-population differences in factors that influence tolerance to oxygen stress [27] were excluded. Space constraints meant that it was unfortunately not possible to replicate this investigation with all populations.

Egg chambers were checked every 24 h and dead eggs counted and removed. When the number of dead eggs in the hypoxic treatment was greater than the control, enough live eggs of the control were extracted to ensure the daily number of eggs removed from each treatment was the same. This ensured that stocking density did not affect treatments unequally.

Processing and analysis of egg architecture and membrane tissue took place three times throughout development when: (1) relative mortality rates (calculated by subtracting mortality rate of the control from the hypoxic treatment) exceeded 5%, 20% and 50% and; (2) the number of eggs that died in a 24 h period was sufficient to provide a robust sample size (≥36 eggs, confirmed using *post hoc* power analysis on the computer program G*power). These thresholds were reached at 134 DDs, 144 DDs and 448 DDs, respectively.

To determine whether membrane structure changed when eggs died, a separate group of 500 farmed eggs were incubated under control conditions. When eggs in this group died (*n* = 37) they were removed within 24 h of their death and sampled alongside the same number of live eggs. Independent samples *t*-tests indicated no structural differences between live and dead eggs (egg diameter: *t*_72_ = 0.209, *p* = 0.835; membrane thickness: *t*_72_ = 0.200, *p* = 0.842; membrane porosity: *t*_72_ = 0.032, *p* = 0.975). This showed membrane structure does not change within 24 h of egg mortality, so validated the methodology.

### 2.4. Processing and analysis of membrane tissue

Before analysing membrane structure, the diameter of each egg was measured under a Nikon E100 microscope at 50× magnification using a scaled background and graticule. To account for measurement error, eggs were re-measured and were within less than 1.5% of the original values. Diameter values were used to calculate the surface area of the egg sphere.

Egg membranes were then prepared for scanning electron microscope (SEM) observation using methods described elsewhere [13], before they were dehydrated in a Balzers 030 Critical Point Drier. Each membrane was fractured into three fragments using forceps to observe the inner membrane surface (internus), outer membrane surface (externus) and cross-section (figure 2). These were affixed to a 12 mm carbon tab on an aluminium stub and sputter coated with gold for 30 s, before inspection under the SEM (Jeol JCM-6000).

Membrane thickness was measured at four locations on the cross-sectional fragment (figure 2*e*) and a mean derived to estimate membrane quotient (*x_p_*) for each egg,

$$x_{p}=\frac{x}{\emptyset },$$

where *x* = mean membrane thickness of individual egg (cm); ∅︀ = mean egg diameter of corresponding population (cm).

The internus was analysed to determine micropore density and diameter because the naturally occurring adhesive film found on the externus [28] (figure 2*f*) obscured images. Pore density, number per egg and mean diameter were calculated following methodology described previously [12] and used to calculate porosity of each egg membrane following the formula,

$$\varepsilon_{\tau }=\;\frac{A_{p}N}{A},$$

where ɛ*_τ_* = membrane porosity (dimensionless); *A*_p_ = mean surface area of micropore opening (cm^2^); *N* = number of micropores per egg; *A* = mean egg surface area of corresponding population (cm^2^).

Finally, membrane permeability to oxygen was calculated according to the formula [25],

$$J=D\varepsilon_{\tau }\left(\frac{C_{o}-\;C_{e}}{x}\right),$$

where *J* = membrane permeability to oxygen (mol cm^−2^ s^−1^); *C*_o_ = required oxygen concentration at Atlantic salmon egg surface at 5°C (2.06 × 10^−7^ mol cm^−3^) to support maximum respiratory requirements; *C*_e_ = oxygen concentration within perivitelline fluid of the eggs (3.34 × 10^−8^ mol cm^−3^).

### 2.5. Statistical analysis

To determine differences in membrane thickness, thickness quotient, porosity and permeability to oxygen among populations, one-way ANOVA tests were performed. Differences in all structural features were compared among the control, dead and live treatments at each mortality threshold using one-way ANOVA tests. When differences among populations or treatments were found, Tukey's *post hoc* tests were performed to see where these differences occurred. In all instances, membrane thickness quotient and porosity data were logit transformed prior to ANOVA analysis. Statistical analysis was conducted using SPSS 21.

## 3. Results

### 3.1. Objective 1: Inter-population variability of egg architecture

**Figure 3.**
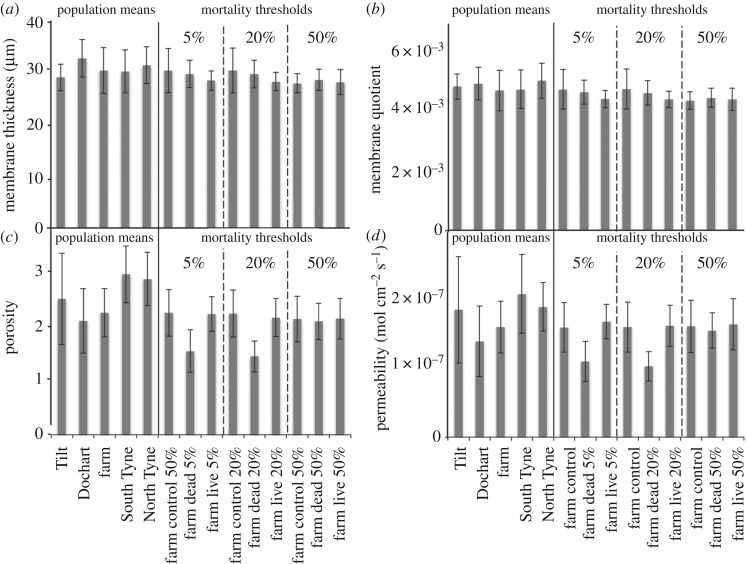
Recorded features of eggs examined under electron microscope relevant to objectives 1 and 2. (*a*) Membrane thickness; (*b*) membrane quotient; (*c*) membrane porosity; (*d*) oxygen permeability. Data on the left of the solid line refer to objective 1 and enable comparison of egg architecture among populations tested. Data on the right of the solid line refer to objective 2 and enable comparison of egg architecture among control eggs and those that died or survived when exposed to hypoxia. Dashed lines separate data for each mortality threshold. Error bars indicate standard deviation.

Differences among populations were observed for egg diameter (*F*_4,176_ = 30.14, *p* < 0.001), membrane thickness (*F*_4,176_ = 4.89, *p* < 0.001; figure 3*a*), porosity (*F*_4,176_ = 14.448, *p* < 0.001; figure 3*c*) and permeability (*F*_4,176_ = 11.941, *p* < 0.001; figure 3*d*), but not membrane quotient (figure 3*b*). River Tilt eggs had a smaller diameter (5.91 ± 0.24 mm) than all other populations (*p* < 0.001) and River Dochart eggs (6.53 ± 0.20 mm) were larger than the farmed (6.36 ± 0.10 mm, *p* = 0.034), South Tyne (6.27 ± 0.33 mm, *p* < 0.001) and North Tyne (6.24 ± 0.44 mm, *p* < 0.001) populations. The River Tilt membranes were thinner than the Dochart (*p* = 0.002) and North Tyne (*p* = 0.045) eggs. The River South Tyne and River North Tyne membranes were more porous than for the River Dochart and Farmed eggs (all comparisons: *p* < 0.001). The membranes of the River Dochart eggs were less permeable than the River Tilt (*p* = 0.001), River South Tyne (*p* < 0.001) and River North Tyne (*p* < 0.001) populations. In addition, eggs of the River South Tyne fish were more permeable than those of the Farmed (*p* < 0.001).

### 3.2. Objective 2: Mass transfer model

**Figure 4.**
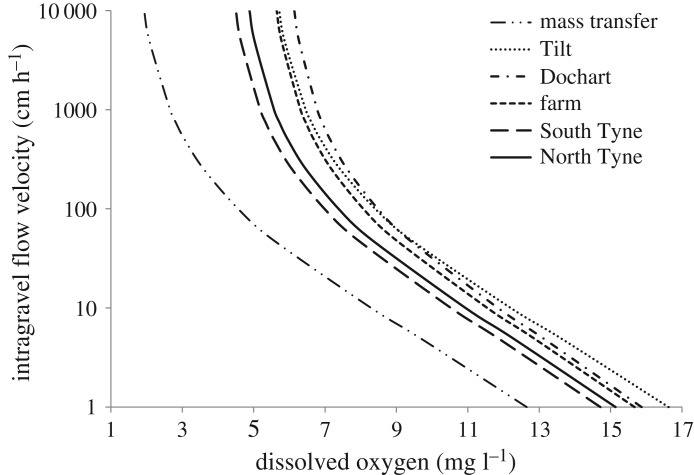
Range of intragravel velocities and oxygen concentrations necessary to support respiratory requirements at 5°C for Atlantic salmon eggs of the five populations investigated in the present study. Data calculated using the mass transfer model (equation (2.1)) and compared with original data for membrane thickness and porosity used in that equation to predict embryonic oxygen requirements. Note that lines do not represent mortality thresholds, but the concentration at which sublethal reductions of post-hatch fitness could be expected.

When the differences in egg structure described above were applied to the mass transfer theory, significant variation in oxygen supply requirements were observed among populations (figure 4). River South Tyne and River North Tyne eggs had lower oxygen supply requirements than the River Tilt, River Dochart and Farmed eggs. The relative requirements of the River Dochart and River Tilt eggs varied depending on whether oxygen or intragravel velocity was limiting. At high intragravel velocity and low oxygen content, River Dochart eggs had higher requirements than the River Tilt eggs. However, the reverse is true at low intragravel velocity but high oxygen concentrations. In addition, estimates of oxygen supply requirements for all populations studied here were substantially higher than those calculated using previous values of membrane thickness (35 µm) and porosity (0.1) taken from [6] and [22] respectively.

### 3.3. Objective 3: Egg structure and hypoxic tolerance of farmed eggs

Comparisons of egg diameter (mean: 6.42 ± 0.12 mm), membrane thickness and membrane quotient at each mortality threshold revealed no differences among treatments (figure 3*a*,*b*). However, there were differences in membrane porosity among treatments at the 5% (*F*_2,97_ = 46.88, *p* < 0.001) and 20% (*F*_2, 102_ = 65.42, *p* < 0.001; figure 3*c*), but not the 50% mortality thresholds. At the 5% and 20% thresholds, the membranes of the eggs that died in the hypoxic treatment were less porous than for the control and those that survived hypoxic conditions (all comparisons: *p* < 0.001). Membrane permeability to oxygen also differed among treatments at the 5% (*F*_2,97_ = 30.913, *p* < 0.001; figure 3*d*) and 20% mortality thresholds (*F*_2,102_ = 36.15, *p* < 0.001; figure 3*d*), but not the 50% threshold. The membranes of the eggs that survived the hypoxic conditions at the 5% and 20% mortality threshold were more permeable than the control treatment and those that died (all comparisons: *p* < 0.001).

## 4. Discussion

While oxygen supply to salmonid ova is a well-known determinant of their survival [4], this study showed egg structure plays a critical role in the incubation success of Atlantic salmon that experience hypoxia. Indeed, differences in membrane structure corresponded with differences in incubation requirements derived using the mass transfer theory and more permeable eggs were more likely to survive periods of oxygen stress. As such, differences in egg membrane architecture could cause variation in hypoxic sensitivity among populations.

Variability of key physical traits among Atlantic salmon populations is well studied in free-swimming life-stages [29], but the differences in egg architecture presented here are the first to show such variation for ova. Egg size is influenced by maternal characteristics such as mass and spawning migration effort [30,31] and environmental conditions such as temperature [32]. However, larger eggs are also likely to have greater rates of oxygen uptake due to a greater diffusive surface area [33], a more permeable egg packet [34] and greater advective flow [35]. The relationship between membrane permeability and oxygen uptake has received less attention, probably due to sampling difficulties. However, studies of other oviparous species [16] have shown that oxygen uptake increases with membrane permeability, so are in agreement with the findings presented here. The relative importance of egg size against membrane permeability in terms of embryonic survival in hypoxia varies according to which factor is limiting oxygen supply. This is particularly evident when applying the mass transfer theory to the River Tilt and River Dochart eggs. At high intragravel velocities the smaller, more permeable River Tilt eggs had lower oxygen requirements than larger, less permeable River Dochart eggs. However, the reverse was true at low intragravel velocities. It is likely that this is because the benefits associated with larger eggs described above means they are better able to compensate for low flow rates. However, as these features are associated with water flow past the egg, they are not likely to be beneficial when concentration is the limiting factor in terms of oxygen supply. While the relationship between egg architecture and oxygen supply is complex, these data imply that the likelihood of Atlantic salmon ova surviving oxygen stress increases with their size and membrane permeability.

In addition to the above findings, the data suggest previous applications of the mass transfer model for Atlantic salmon (e.g. [36]) overestimated membrane porosity. In particular, a porosity value of 0.1 derived from visual estimates of SEM images of chum salmon (*Oncorhynchus keta*) egg membranes [25] has, up to now, been the basis of estimates of oxygen delivery to incubating embryos for all salmonid species. By using high-resolution imagery and accurate measurement apparatus, this study demonstrated this value was 71–79% greater than Atlantic salmon ova porosity. Therefore, estimates of oxygen supply to salmonid embryos should be re-adjusted to represent these updated values, which could have important implications for catchment management practices [19,20]. In addition, the importance of inter-population variability should also be considered in future oxygen supply estimates.

The hypoxic tolerance investigation supported the hypothesis that the tolerance of Atlantic salmon ova to oxygen stress increases with greater membrane permeability. This is in agreement with work on other oviparous species that demonstrates more permeable eggs are better able to tolerate periods of hypoxia [37,38]. The observation that there was no difference in egg diameter between embryos that died and those that survived low oxygen conditions implied membrane permeability is a greater determinant of the sensitivity of Atlantic salmon ova to hypoxia than size. However, it is important to note that the standard deviation of egg size values (4.6%) was substantially lower than membrane permeability (24.0%). As such, differences in diffusive surface area of egg membranes in this investigation was primarily driven by variance of permeability not egg size. Therefore, if egg size variation had been greater, different conclusions may have arisen. Indeed, a study [33] that investigated a greater range of sizes in brown trout found eggs that were on average 37% heavier than conspecifics were more likely to survive periods of hypoxia. This suggests that the narrow range of sizes tested in the present study could have masked the importance of egg diameter in terms of the sensitivity of Atlantic salmon embryos to hypoxia. Nevertheless, the findings provide strong evidence that a range of egg structural factors influence the sensitivity of incubating Atlantic salmon embryos to hypoxia.

While this study clearly shows the structure of Atlantic salmon ova varies among populations, and tolerance to oxygen stress increases with membrane permeability, it does not confirm this variation is adaptive to natural spawning conditions. Nevertheless, adaptation to low oxygen supply is widespread throughout the animal kingdom [39]. Furthermore the permeability of Peruvian coot eggshells negatively correlates with oxygen availability among populations [16]. Therefore, the variety of incubation conditions that Atlantic salmon ova experience throughout the species' range could give rise to structural adaptation. However, this requires further investigation.

The data presented in this study cannot provide conclusive evidence that Atlantic salmon egg structure varies as part of an adaptive compensatory response to different exposure to hypoxic stress. However, the findings could have important implications for commonly used stock enhancement techniques. If genetic adaptation is demonstrated in future studies, then interbreeding between native and non-native individuals as a result of translocation [40] or interactions with escapees from salmon farms could drive the loss of beneficial egg structural traits [41]. Furthermore, while supportive breeding, which involves rearing eggs of wild salmonids in a hatchery environment before returning juveniles to their native streams, does not have the same direct genetic influence, it reduces selective pressure on the egg stage. Although low mortality in the hatchery environment means selection-driven loss of low oxygen adaptation is unlikely [42], the process can lead to loss of genetic variability and phenotypes beneficial to the natural habitat [43]. As such, this study adds to the growing body of evidence that supplementary breeding techniques could have long-term negative consequences for salmonid populations (e.g. [43]), so should be used with caution.

For the first time, this study demonstrated inter-population variability of teleost egg structure that is likely to play a key role in the interactions between the embryo and its environment. While it is not possible to conclusively demonstrate this variability is adaptive in nature, there is convincing evidence that structural variation among populations will influence embryonic oxygen supply requirements and tolerance to oxygen stress. If further research demonstrates this variation is adaptive, there are important implications for management techniques that influence gene flow among populations and incubation conditions experienced by wild populations.

## Data accessibility

Our data are deposited at the University of Southampton data repository: https://eprints.soton.ac.uk/425565/.
